# Epidemiologic features of neonatal sepsis and its COVID-19 associated temporal patterns in Jimma Medical Center, Ethiopia: A Joinpoint regression analysis

**DOI:** 10.1371/journal.pone.0291610

**Published:** 2023-11-02

**Authors:** Daniel Geleta, Gemeda Abebe, Netsanet Workneh, Getenet Beyene

**Affiliations:** 1 Department of Medical Laboratory Sciences, Jimma University, Jimma, Oromia, Ethiopia; 2 Department of Pediatrics and Child Health, Jimma University, Jimma, Oromia, Ethiopia; JABSOM: University of Hawai’i at Manoa John A Burns School of Medicine, UNITED STATES

## Abstract

**Background:**

Neonatal sepsis remains a leading cause of morbidity and mortality in neonates across all regions, including Africa. Compared to developed and some developing countries, there are relatively few epidemiological trends for neonatal sepsis and associated patterns with COVID-19 in Ethiopia. We modeled an epidemiological trend and pattern to aid in the monitoring of changes in neonatal sepsis.

**Methods:**

Retrospective data were collected from all admissions to the Neonatal Intensive Care Unit (NICU) in Ethiopia at Jimma Medical Center between May 2019 and April 2022. We analyzed the monthly neonatal sepsis incidence, mortality, and case-fatality rates using STATA software. Finally, we modeled a monthly time series of neonatal sepsis incidence trends and patterns associated with the COVID-19-impacted period using Joinpoint software. For all analyses, a P value of 0.05 was considered statistically significant at the 95% confidence interval (CI).

**Results:**

In the 36 months, 6796 cases were admitted to the NICU, with a 9.5% (95% CI: 9.1, 10.0) incidence rate of neonatal sepsis. The overall admission mortality rate was 16.5% (95% CI: 13.6, 19.8), while sepsis-attributed mortality was 7.1% (95% CI: 5.8, 8.5). The data showed an unstable decreasing trend for three Joinpoints (August 2020, December 2020, and August 2021). Notably, a decrease in the incidence trend was observed from May 2019 to August 2020 (MPC, -4.1; 95% CI: -7.6, -0.5; P = 0.03), followed by a sharp increase (MPC, 23.7; 95% CI: -13.8, 77.7; P = 0.24) from August 2020 to December 2020. From December 2020 to August 2021, there was again a decreasing trend (MPC, -13.8; 95% CI: -23.3, -3.5; P = 0.01), followed by a slight increase from August 2021 to April 2022 (MPC, 4.2; 95% CI: -8.4, 18.6; P = 0.52). Finally, the study revealed an association between patterns of neonatal sepsis incidence trends and COVID-19, with a Joinpoint jump model comparability ratio (CR = 0.43) between pre- and COVID-19-impacted periods.

**Conclusions:**

Neonatal sepsis was prevalent at Jimma Medical Center, but it was on an unstably declining trend. The current results suggest a potential temporal association between the intensity of COVID-19 containment measures and a change in the incidence trend and patterns of neonatal sepsis. However, the quantified contribution of a particular containment measure requires further investigation.

## Introduction

Neonatal sepsis is a life-threatening medical condition [1, 2] that makes the neonatal period the most perilous time for child survival [3]. Global initiatives that include research, innovation, service delivery programming, and advocacy are being used to combat it [4]. However, preventable daily tragedies continued to occur around the world [5, 6], notably in middle- and low-income countries [2], due to haphazard initiatives [4] and the non-monotonic nature of neonatal sepsis [1, 7].

Epidemiological statistics reveal that the incidence of neonatal sepsis varies by region, most notably by country [8, 9]. It appeared to be four to ten times higher in developing countries than in developed countries, and it was mostly unknown in the majority of developing countries [10, 11]. It ranked among the top 10 causes of mortality in 2014, with no significant change to date [10]. In 2019, it caused the most morbidity in South Asia and sub-Saharan Africa [12]. Its overall incidence in the United States [13], Indonesia [12], Tanzania [14], Kenya [15], and Ethiopia [16] was estimated to be 3.1%, 5%, 23.9%, 29.1%, and 11 in 1000 live births, respectively. Concurrent reports also revealed in-hospital mortality rates of 18.2% in Kenya [15], 28.3% in Indonesia [12], and 17.8% in Ethiopia [17]. Specifically in Ethiopia, the neonatal mortality rate has spiraled by 4% in the last three years and remains high at 33 per 1000 live births [18]. In a similar vein, each country has experienced a different percentage change in the incidence of neonatal sepsis. The percentage change in the United States and Kenya has dropped by 3.6%, according to the literature, but no incidence trend data for Ethiopia were available at the time of our review [13, 15].

Fascinatingly, the percentage change in neonatal sepsis rates varies by transition point, varying across distinct time segments due to various reasons [13, 19]. The COVID-19 pandemic, for example, has had severe and far-reaching repercussions for health systems, strained families and communities [20], and impeded joint neonatal action plans [21]. It has then decreased the utilization of basic maternal, neonatal, and children’s health care services [22] and poignantly sparked a new normal for neonatal mortality in developing countries [2, 22, 23]. Studies revealed the pandemic has positively impacted the epidemiology of neonatal sepsis in India [24], Bangladesh, Nigeria, South Africa [25], Saudi Arabia [26], Malawi, and rural Uganda [27], but had no significant impact in China [28] and Turkey [29]. Likewise, the impacts of the COVID-19 stringent index on the epidemiologic features of neonatal sepsis were not well documented in these countries and were largely unknown in the neonatal population, including Ethiopia [23, 30, 31].

Given the recent fragility of the epidemiology of neonatal sepsis, the authors of this article intended to model it using data from hospitals that thought to be free of recall bias [14] and to underline its relationship with COVID-19-impacted periods in Ethiopia. In Ethiopia, the national-wide lockdown policy was launched in April 2020 but gradually lifted [28, 32, 33] and replaced by the COVID-19 government’s stringent index or containment measures (neonatal visiting limitations, wearing a face mask, effective hand washing, physical distancing, and vaccination) [23, 30, 31]. These containment measures were announced in mid-March 2020 and widely publicized in April 2020 in Ethiopia [34].

## Materials and methods

### Study design, setting and period

The researchers used retrospective data to model the epidemiologic features of neonatal sepsis at Jimma Medical Center (JMC), a tertiary and referral teaching medical center in the Oromia National Regional State in Ethiopia. The center serves 15 million outpatients and 16,000 inpatients each year, with various departments operating 24 hours a day. One of its departments, the pediatric department, comprises a separate neonatal intensive care unit (NICU) [35]. The unit contains 53 beds (6.6% of JMC bed capacity) and admits an average of 75 neonates per month for various complaints. It provides various medical services, including thermoregulation, infection control, nasogastric feeding, intravenous hydration, phototherapy, blood transfusion, basic laboratory tests, oxygen therapy, and pulse oximetry.

The center used a clinical approach that followed the criteria of clinical sepsis to establish the diagnosis of neonatal sepsis. These criteria included fever (> 38°C) or hypothermia (< 36°C), rapid breathing (>60 breaths/minute), severe chest indrawing, poor feeding, seizure, lethargy, or unconsciousness, and two hematologic criteria: total leukocyte count 5000 or >12,000 cells/m3, absolute neutrophil count 1500 cells/mm3 or >7500 cells/mm3, ESR >15/1 h, and platelet count 150,000 or >440,000 cells/mm3) [36]. Only blood culture tests were available and were then used to confirm the presence of sepsis. Six to eight nurses are on duty each shift to care for the neonates, along with three to four assigned resident doctors and two pediatricians who round on the patients and are available on call every day. Water is piped into the structure, and a standby generator makes electricity available round-the-clock [37].

The records of individual patients at the unit were recorded daily on the patient chart, summarized in a logbook at discharge, and reported monthly to the Health Information Management System (HIMS) office. The HIMS office then transfers and stores the data using the District Health Information System 2 (DHIS2) software. Since September 2019, the HMIS office of the center has used a DHIS2 database to store the profile of every patient by demography, diagnosis, treatment, and the treatment outcome of each hospitalized patient [35]. The data for this study included the immediate pre-COVID-19 periods and COVID-19-impacted periods, which ranged from May 2019 to April 2022.

### Data variables, data collection and data source

Data variables included: (i) all cases admitted to the NICU aged less than 28 days; (ii) the number of treated neonatal sepsis cases identified by clinicians each month; and (iii) the number of related mortalities each month. The majority of the data was generated from DHIS2 NICU monthly reports, with only four months of data sourced from hospital-based patient records (logbooks) via a validated tool from March 1–15, 2022. The data collection process began after the Jimma University Institute of Health Science Review Board approved the research protocol [with reference number JUIRB32/2022 on February 09, 2022], and the Jimma Medical Center Ethical Committee waived the requirement for informed consent [with reference number THRPGn/344/2022 on February 22, 2022]. Further, all neonatal data were fully anonymized before being accessed during data collection.

### Participant selection and sampling

**Fig 1:**
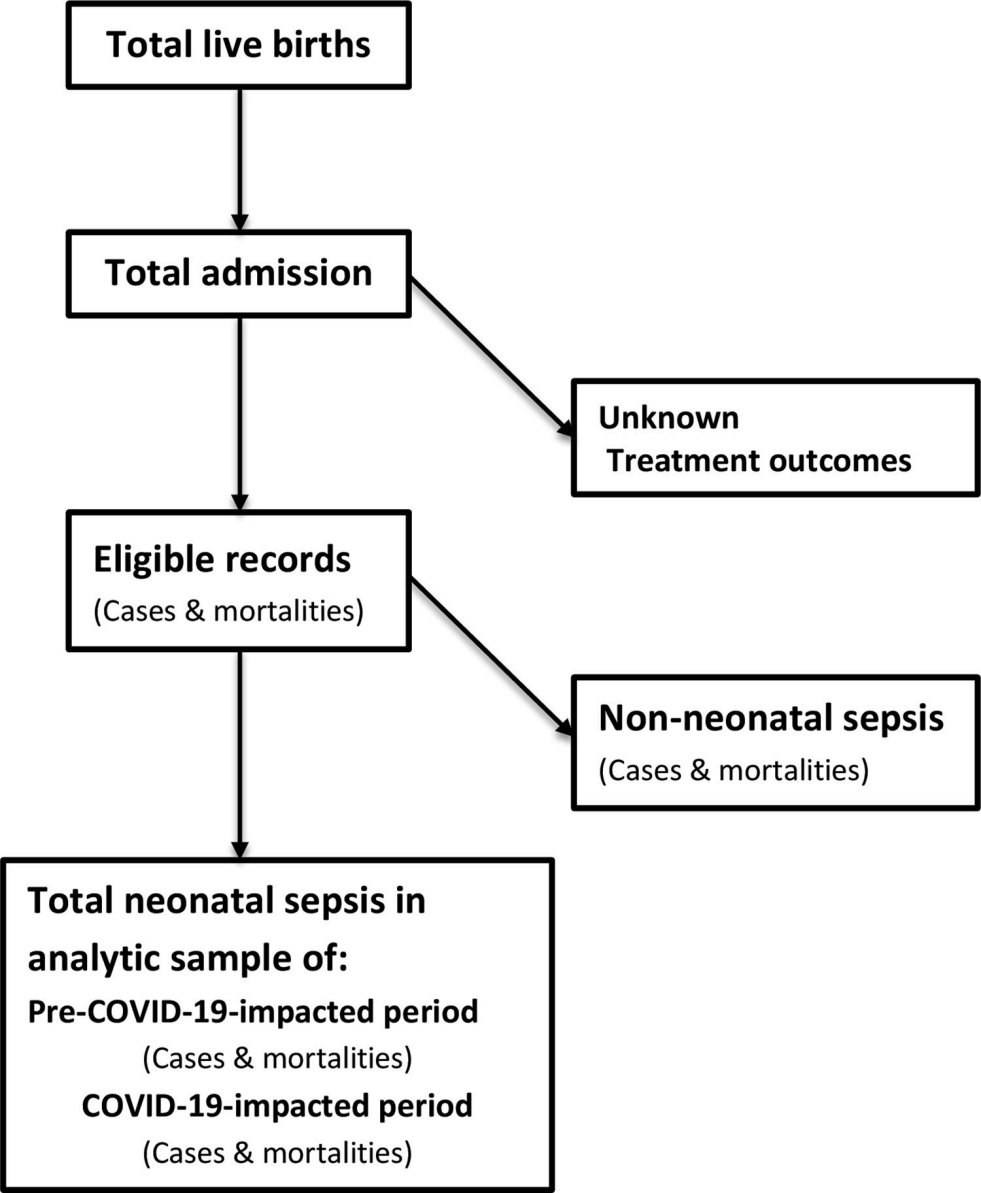
Study participant selection and distribution flow-chart.

We analyzed all neonatal sepsis cases in live births diagnosed by clinical, biomarker, culture, or combination methods. The study included only inborn neonates at JMC with complete records and known treatment outcomes; otherwise, they were excluded (Fig 1).

### Outcome measures

The authors estimated the overall incidence, admission, and case-fatality rates of neonatal sepsis. Furthermore, they presented the trend using a rate of monthly percent change for the incidence of neonatal sepsis. We also explained the pattern of the trend of neonatal incidence under the impact of COVID-19 containment measures, assuming April 2020 as a transition point or the start of impacted period. The Ethiopian government issued countrywide COVID-19 containment measures for the public health problem for the first time on April 8, 2020, and JMC began executing the measures the same month [38].

### Statistical analysis

The data from paper-based extraction was entered into DHIS2 and exported into appropriate software with the already available DHIS2 data. The authors estimated the overall incidence rate of neonatal sepsis by dividing the number of neonates with sepsis by the total number of live births. In contrast, the neonatal sepsis mortality rate was calculated using all neonatal deaths that occurred during the study period as a nominator and live births as a denominator. Finally, the admission mortality and case fatality rates were estimated from confirmed deaths of neonatal sepsis to the admitted neonatal population and deaths due to sepsis, respectively. The confidence interval of all rates was calculated using the Poisson distribution, and all rates were calculated in hundreds. We conducted a stepwise analysis to appraise the features of the incidence rate at each time portion. After testing for trends using the Mann-Kendall test, we initially fitted the rates into a sequential model to identify the conventional trend that also identified the months with the lowest trough and the highest peak using STATA software version 16.0.

Secondly, the monthly percent change was modeled using Joinpoint trend analysis software version 4.9.1.0. For modeling, we considered incidence rate as the dependent variable and incidence months as the independent variables. In this particular model, the number of sepsis cases is used as a numerator, while the number of live births is used as a denominator. For the MPC analyses, a series of Monte Carlo permutation tests were used to select the final model [19]. Finally, we examined and compared the patterns of the trends of neonatal sepsis in a binary time frame against the impact of COVID-19 using a jump model. The binary time period was defined as a pre-COVID-19 period ranging from May 2019 to April 2020 and a COVID-19-impacted period ranging from May 2020 to April 2022. For all analyses, a P-value of 0.05 was considered statistically significant at the 95% confidence interval.

## Results

### Enrolment and distribution of study participants

The study successively selected and distributed participants within a 36-month time period, as depicted in Fig 1. It enrolled 16,923 live births at JMC, with 6812 (40.3%) of them admitted to NICU before their 28^th^ birthday. Of the admitted, 6796 (99.8%) records met inclusion criteria and provided data for the study, and 698 (10.3%) of them documented for mortality attributed to all causes. The majority of recorded cases, 5187 (30.7%), were non-neonatal sepsis cases, with 583 (11.2%) having mortality records. Nearly a quarter of the admitted cases, 1609 (23.7%), were neonatal sepsis, with 115 (1.7%) mortalities reported from the condition. The male sex accounted for 59.7% of total admissions, 59.0% of neonatal sepsis cases, and 58.3% of sepsis-related deaths, while the female sex contributed the remaining proportion. The majority of total admissions (68.2%) and over half of all recorded neonatal sepsis (62.4%) occurred during the COVID-19-impacted period (Table 1).

**Table 1:** Distribution of neonatal sepsis before and during COVID-19-impacted period at JMC, Ethiopia.

Cohorts	Neonatal sepsis: n (%)			Non-neonatal sepsis: n (%)		AMR: n (%)	Total admitted N (%) *
	Incidence	PMR	CFR	Incidence	PMR		
**Total live birth (16923)**							
Male	949(59.0)	67(58.3)	4.2	3111(60.0)	345(59.2)	412(59.0)	4060(59.7)
Female	660(41.0)	48(41.7)	3.0	2076(40.0)	238(40.8)	286(41.0)	2736(40.3)
**Subtotal**	1609(9.5)	115(16.5)	7.1	5187(30.7)	583(83.5)	698(10.3)	6796(40.2)
**Caseload before the COVID-19-impacted period; Live births: 5301(31.3)**							
Male	350(57.9)	21(72.4)	3.5	943(60.6)	115(60.8)	136(62.4)	1293(59.8)
Female	255(42.1)	8(27.6)	1.3	613(39.4)	74(39.2)	82(37.6)	868(40.2)
**Total**	605(11.4)	29(13.3)	4.8	1556(29.4)	189(86.7)	218(10.1)	2161(31.8)
**Caseload during the COVID-19 impacted period; Live births: 11622(68.7)**							
Male	599(59.5)	46(53.5)	4.6	2168(59.7)	230(58.4)	276(57.5)	2767(59.7)
Female	405(40.5)	40(46.5)	4.0	1463(40.3)	164(41.6)	204(42.5)	1868(40.3)
**Total**	1004 (8.7)	86(17.9)	8.6	3631(31.3)	394(82.1)	480(10.4)	4635(68.2)

PMR, Proportionate Mortality Rate; CFR, Case-fatality Rate; AMR, Admission Mortality rate

*after exclusion of 16 records of unknown treatment outcome

### The overall key parametric and temporal features of neonatal sepsis

The incidence rate of neonatal sepsis was 9.5 (95% CI: 9.1, 10.0) per 100 live births, resulting in 16.5% (95% CI: 13.6, 19.8) admission mortality and 7.1% (95% CI: 5.8, 8.5) case-fatality rates. Male neonates accounted for 4.1% (95% CI: 3.3, 5.3) of the total case-fatality rate, while females accounted for 3.0% (95% CI: 2.2%, 4.0) (Table 1). Similarly, the monthly incidence rate showed an erratic trend with nine peaks and eight troughs, with the highest surge (18%) occurring in December 2020 and the lowest (3%) in October 2021. This naive model, without a Joinpoint, showed a statistically significant (test statistic = 0.33, P < 0.01) decrease from 11.5% (95% CI: 14.6, 22.1) to 4%95% CI: 1.5, 5.2).

**Fig 2:**
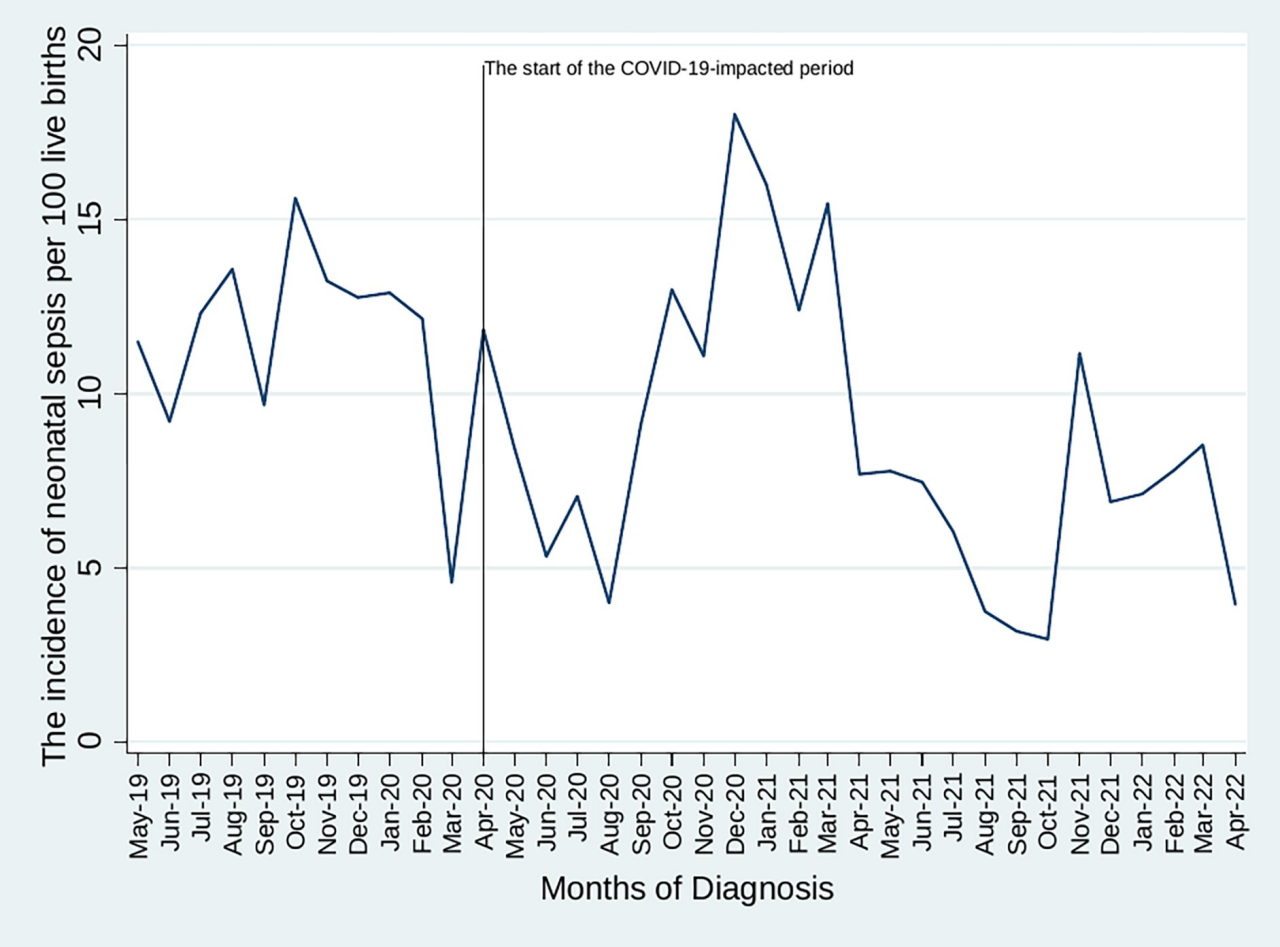
The incidence rate of neonatal sepsis among neonates at JMC, Ethiopia.

**Fig 3:**
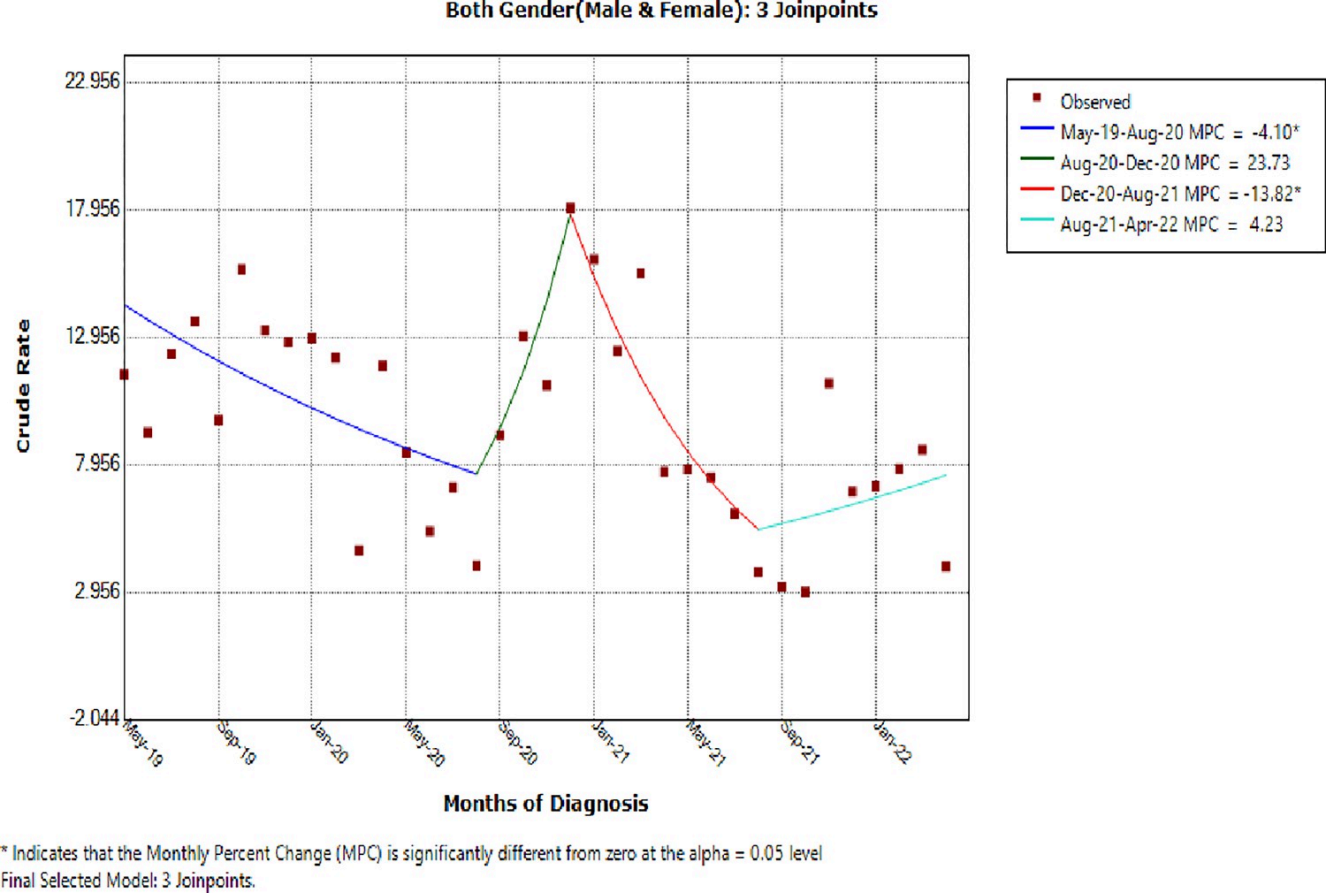
The monthly percent change of neonatal sepsis incidence trend at JMC, Ethiopia.

Based on the data in Fig 2, the trend was modeled, and three Joinpoints were identified (August 2020, December 2020, and August 2021), indicating that the trend line changed three times (Fig 3).

Accordingly, in Fig 3, there was a statistically significant (P = 0.03) decrease in the trend of neonatal sepsis incidence between May 2019 and August 2020 (MPC, -4.1; 95% CI: -7.6, -0.5). However, there was a sharp increase (MPC, 23.7; 95%CI: -13.8, 77.7) between August 2020 and December 2020, with no statistical significance (P = 0.24). There was again a decreasing trend between December-2020 and August-2021 (MPC, -13.8; 95 percent CI: -23.3, -3.5), with a statistically significant rate (P = 0.01), followed by a non-significant increase (P = 0.5) between August 2021 and April 2022 (MPC, 4.2; 95% CI: -8.4, 18.6). The estimated regression coefficients (beta) from general parameterization were -0.04 for slope 1 (p = 0.03), 0.21 for slope 2 (p = 0.24), -0.15 for slope 3 (P = 0.01), & 0.04 for slope 4 (Table 2).

**Table 2:** Neonatal sepsis incidence trend variations by period and MPC at JMC, Ethiopia.

Cohorts	MPC Parameters at 95%CI				
	Time Segments	Change Months (Endpoints)	Parametric estimate(B)	%IR (95%CI)	P-Value
Sex (Male & Female)	(1)	May-2019 to Aug-2020	-0.04	-4.1*(-7.6, -0.5)	0.03*
	(2)	Aug-2020 & Dec-2020	0.21	-13.8(-23.0, -3.5)	0.24
	(3)	Dec-2020 & Aug-2021	-0.15	-13.8*(-23.0, -3.5)	0.01*
	(4)	Aug-2021 & Apr-2022	0.04	4.2(-8.4, 18.6)	0.52

MPC, Monthly Percent Change

* Indicates significance at P-Value of 0.05

#### Incidence patterns of neonatal sepsis at the COVID-19-impacted period

**Fig 4:**
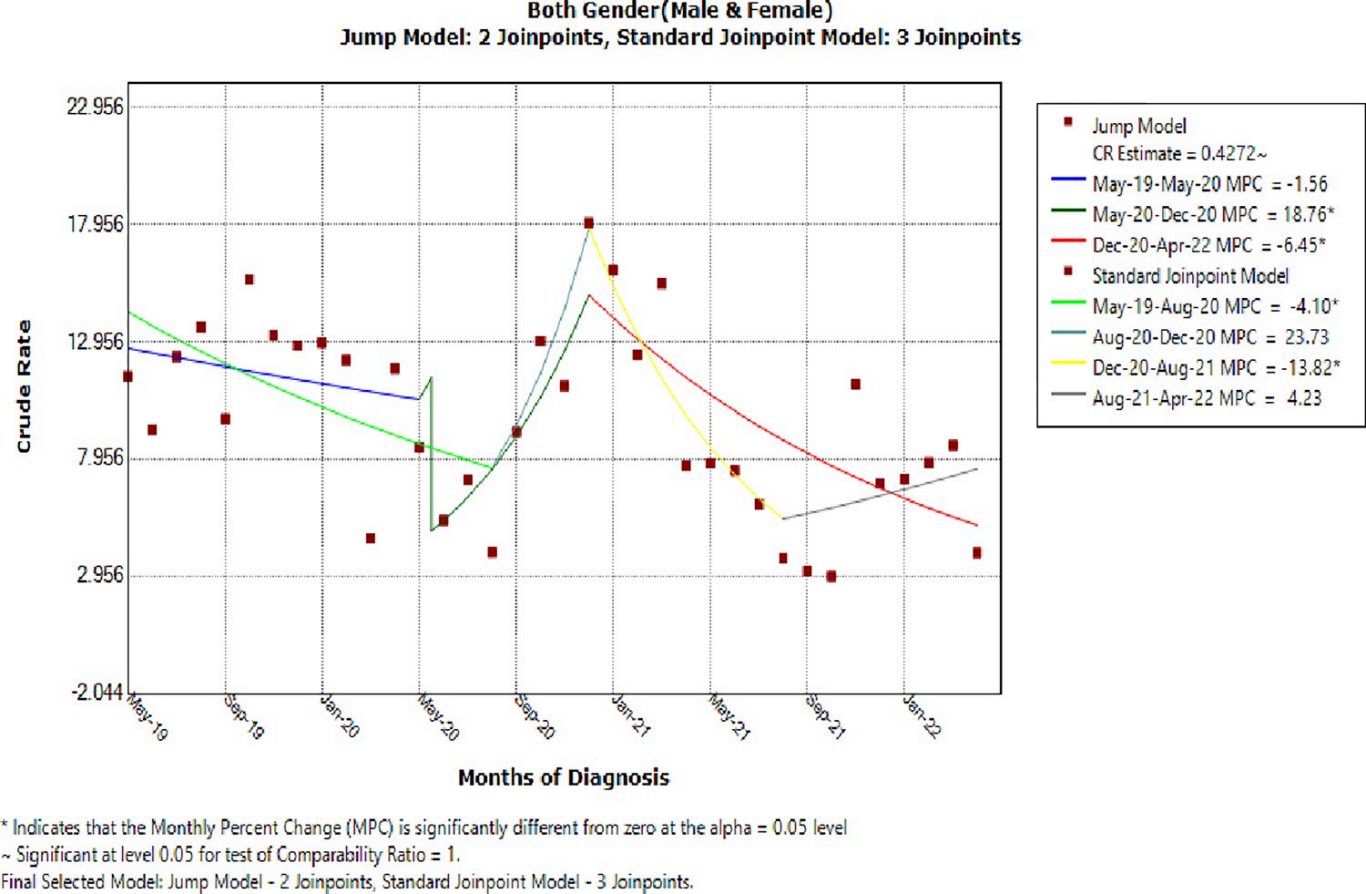
The overall CR jump model for neonatal sepsis incidence rate, JMC, Ethiopia.

Neonatal sepsis was more common before the COVID-19 impacted period, at 11.4% (95% CI: 10.5, 12.3), than it was within COVID-19 time, at 8.7% (95% CI: 8.1, 9.2). The sepsis-related mortality rate was 13.3% (95% CI: 8.9, 19.1) before the impacted period and 17.9% (95% CI: 14.2, 21.7) during the impacted period. However, the pre-COVID-19 mortality rate was 10.3%10.3% (95% CI: 9.5, 11.3), and remained roughly the same during the impacted period. The case-fatality rate of neonatal sepsis was 4.8% (95% CI: 3.2, 6.9) during the pre-COVID-19-impacted period and 8.6% (95% CI: 6.7, 10.6) during the impacted period. Comparing trends in neonatal sepsis incidence between pre- and during COVID-19 impacted periods yielded an estimated comparability ratio (CR) value of 0.43, indicating a declining statistically significant value (Fig 4).

Furthermore, the MPC patterns for each model segment or time period were identified. Accordingly, the trend in the incidence of neonatal sepsis showed an MPC of -1.6% (95% CI: -6.4, 3.6), with no statistically significant decrease from May 2019 to May 2020 (P = 0.5). Whereas, the MPC was 18.8 (95% CI: 1.7%, 38.7) between May 2020 and December 2020, indicating a statistical significance increase (P = 0.03), and finally, the MPC was -6.5 (95% CI: -9.6, -3.2) between December 2020 and April 2022, indicating a statistical significance decrease (P = 0.01). From the general parameters, the estimated regression coefficients (beta) were -0.02 for slope 1 (P = 0.5), -0.17 for slope 2 (P = 0.03), and -0.07 for slope 3 (P = 0.01) (Table 3).

**Table 3:** A Jump model for neonatal sepsis incidence trend patterns at JMC, Ethiopia.

Cohorts	MPC Parameters at 95%CI				
	Time Segments	Change Months (Endpoints)	Parameter estimate(B)	%IR (95%CI)	P-Value
**Sex (Male & Female)**	(1)	May-2019 to May-2020	-0.02	-1.6(-6.4, 3.6)	0.5
	(2)	May-2020 & Dec-2020	0.17	18.8(1.7, 38.7)	**0.03** *
	(3)	Dec-2020 & Apl-2022	-0.07	-6.5(-9.6, -3.2)	**0.01** *

MPC, Monthly Percent Change

* Indicates significance at P-Value of 0.05

## Discussion

We reviewed the epidemiologic features of neonatal sepsis in the JMC of Ethiopia using a Joinpoint analysis, which was supported by previous research to detect change points in trends. The review found that neonatal sepsis is a common reason for admission to NICU and that it has been decreasing in recent months. The overall decreasing incidence trend, however, was not stable, and the decreasing pattern was associated with COVID-19 containment measures in the study area.

Specifically, the current study revealed an incidence rate of 9.5%, an admission rate of 23.7%, a neonatal mortality rate of 16.5%, and a neonatal sepsis mortality risk of 7.1% over a 36-month period. This incidence rate is nearly consistent with the estimate of the global incidence rate in low- and middle-income countries. But it is higher than the incidence rate reported in the United States by more than two-fold [13] and higher than the rate reported in India by 3% [39]. Conversely, the recent study’s incidence rate is lower than incidence rates of 30.5% in Korea [40], 23.9% in Kenya [15], 89.3% in Iran [41], and 49.5% in Egypt [42]. Likewise, the current result mortality rate of 16.5% is commensurate with the global mortality rate of 17.5% [10], but it was found to be significantly lower than the mortality rates of 24.7% in Iran [41], 29.1% in Tanzania [14], and 23.1% in another area of study result in Ethiopia [43]. The 16.5% attributed mortality rate in this study, on the other hand, is higher than the 15% WHO global estimate [44],12.6% in China [41], 15.8% in South Asia study reports [12] and 12% 2030 global action plan [45]. Further, the 7.1% chance of dying, or CFR, in this study outweighs the South Korea’s 2.2% CFR and Nigeria’s infrequent CFR reports [46]. The CFR of our study, however, is lower than the 18% pooled global CFR [47] and the 18.2% of Kenya [15]. Finally, the CFR of this study is consistent with the approximately 7% CFR reports of Taiwan and Asia [41, 48].

Several hypotheses exist about the cause of the variation in epidemiologic features of neonatal sepsis. The most common causes of variation are related to the level of care-seeking behavior, diagnostics, sepsis management, and the health status of the neonates [49]. In countries with substandard neonatal care, like Ethiopia, neonates are consistently predisposed to neonatal sepsis risk factors. For example, the risk of dying from neonatal sepsis is 2% in term infants, 20% in preterm infants, and 30% in those with co-infection [47]. Practically, some facilities, including the current study area, fail to provide the simplest immediate breastfeeding after delivery, possibly increasing the aforementioned proportion and resulting in variation in the incidence rates of neonatal sepsis [50]. In addition to these factors, the rise in antibiotic resistance, changes in healthcare-seeking behavior, the growth of invasive procedures, reduced exposure to infectious agents, change in hygiene practices, the use of different denominators by researchers when calculating neonatal sepsis incidence, and the absence of preventive measures for *E*.*coli* in newborn sepsis all play a role in the variations and instability in the estimates of neonatal sepsis [11].

We were also able to locate the change points and their direction in time when the incidence trend of newborn sepsis considerably changed. The short-term trend indicated a statistically significant decrease of 4.1% from May 2019 to August 2020, followed by a non-statistically significant rise of 13.8% from August 2020 to December 2020. It showed a statistically significant decline of 13.8% in the second to last section between December 2020 and August 2021, followed by a non-statistically significant increase of 4.2% thereafter. In this instance, the last segment was not statistically significant despite the preceding declining trend being statistically significant [19].

Despite the lack of methodologically comparable and sufficient studies to compare with, the current finding suggests a reasonable and pressing change. Because the current trend analysis revealed upward and downward moving trends, indicating an unstable incidence trend. The feature of the current study’s finding is a global feature and a concern as well, as seen in Tanzania [14]. Though the magnitudes of the average percentage change vary, the current incidence trend is in the same direction as the declining percentage changes in Brazil [51], India [39], the United States, and the 2020 global report [13, 52]. Of note, the current study result implies an inconsistent decline in the incidence trend, necessitating close monitoring of the progress toward the global neonatal sepsis initiatives in the study area. However, the variation in magnitude and direction of the average percentage change could be explained by the ongoing intrapartum and postnatal infection-control practices and type of diagnostic used [4].

Some countries endorse maternal antisepsis, such as vaginal chlorhexidine during labor and antibiotic prophylaxis during delivery, to significantly reduce the incidence trend of neonatal sepsis. whereas, regrettably, middle- and low-income countries do not use any of the prevention methods or use them inconsistently [53, 54]. Evidence also points to a clear relationship between the incidence trend of newborn sepsis and the intensity of the COVID-19 containment measures. Despite several confounding variables, this finding is similar to the lesson learned in India, where strict adherence to COVID-19 containment measures resulted in a 1.19% decrease in the incidence trend of neonatal sepsis [55]. More importantly, compliance levels in the center were variable during intense periods, and they eventually diminished with the pace of the pandemic [56], likely contributing to the instability of neonatal sepsis incidence trends [57, 58].

Before the COVID-19 pandemic, neonatal sepsis was already a significant concern in healthcare settings, including JMC. The observed declining incidence trend in the first joinpoint could be the result of the concern. However, during the period of the second joinpoint, there was a sharp increase of 23.7% in MPC from August 2020 to December 2020. In this period, JMC and many healthcare facilities under the JMC catchment experienced disruptions, reduced capacity, or focused primarily on COVID-19 response. In the period, JMC applied COVID-19 containment measures following the initial wave of the pandemic and led the containment activities in the center, including screening areas, testing centers, isolation wards, physical distancing, visitor restrictions, sanitization, and strict protocols for personal protective equipment. The center limited visitors to essential caregivers, screened them, and provided physical distancing in waiting areas, cafeterias, and common areas, particularly in the delivery ward and NICU (59). The period in JMC, moreover, was a complete lockdown and could be hypothesized to have caused reduced routine postnatal visits (both at home by community-based health care workers and in health care facilities) and often resulted in a rise in the incidence of neonatal sepsis.

During the second joinpoint, however, the complete lockdown was lifted and health facilities reopened with stringent COVID-19 indices. During the reopening of JMC, strong COVID-19 infection prevention and control measures were put in place. Some measures, such as screening pregnant women for infections, appropriate use of personal protective equipment (such as masks, gloves, gowns, and eye protection), improved hygiene practices (regular handwashing with soap and water, using hand sanitizers at the bedside of each individual neonate, no handshake, and daily ward cleaning), and limited health professionals on duty shift, were implemented and might have contributed to a reduction in the incidence of neonatal sepsis by 13.8%. Lastly, the epidemiology of COVID-19 faded, and its stringent measures changed over time and began to relax. In JMC during the third joinpoint period, just a few regular infection prevention and control procedures were observed, conceivably contributing to the resurgence of neonatal sepsis [57, 59].

The incidence, mortality, and case-fatality rates of neonatal sepsis did not differ significantly between the pre-COVID-19 and COVID-19-impacted periods. The incidence trend between these time periods, on the other hand, revealed a statistically significant decrease in both the jump and standard Joinpoint models. The Joinpoint-jump model, which was designed to simultaneously measure the size of the jump and the changes in trend, confirmed a significant value of comparability ratio (CR) less than one (the estimate of the comparability ratio statistically different than 1), whereas the standard Joinpoint model remained unaffected. The comparability ratio model provides a direct estimation of trend data where there is a systematic scale change, which causes a "jump" in the rates but is assumed not to affect the underlying trend. It implies if the change occurred among cases recorded under certain old and new systems, which effectively means the ratio of the case counts after the change to before the change. The new system has no effect on the total net count with a CR of 1, but classifies fewer events with a CR below 1 and more with a CR greater than 1 [60]. Therefore, based on the comparability ratio of our jump model, we can conclude that the incidence rate of neonatal sepsis was lower during the impacted period than during the pre-COVID-19 period [32].

In more detail, the Jointpoint-jump model initially found the first Joinpoint at May 2020, with a non-significant drop of 1.6% per month prior to the Joinpoint, and the second Joinpoint at December 2020, with a significant rise of 18.8% per month prior to the Joinpoint and a significant drop of 6.5% per month after. For trend analysis, the last segment tends to draw the most recent interest because of its important indication of the most recent trend. In this regard, the current graph of the Joipoint-Jump model has demonstrated a downward trend in two of its last four segments. Therefore, it could be likely to conclude that COVID-19 has influenced the pattern of incidence trend of neonatal sepsis to decline with a particular trend. The current decline in incidence trend is consistent with a significant decline in China [28], India [24], and Rwanda [32], unlike Italy, where there was no change between the pre-COVID-19 and COVID-19 impacted periods [61].

For the reason of discrepancies among the results, a multi-faceted factor could be hypothesized, while strict visitation policies and health-seeking behaviors take the principal role [53]. Because of the frustration from COVID-19 and sepsis overlapping symptoms, neonatal caregivers’ health-seeking behaviors appeared to be at the earliest possible time during COVID-19, contributing to a variable degree of reduction in the features of neonatal sepsis [62]. Of note, Hospital-based deliveries during the COVID-19 pandemic have not decreased, but were attended by a limited number of relatives and with senior clinicians on duty. This could indicate a reduction in trafficking at the delivery ward and NICU, contributing to reduced neonatal sepsis [33]. Most importantly, the components of the COVID-19 government stringent index exactly match the protective measures recommended by pediatricians for neonate prevention against sepsis. Consequently, optimal neonatal visitation restrictions, wearing a face mask at all times when in contact with the neonate, effective hand-washing before and after caring for the neonate, and physical distancing could all help to decrease the incidence trend of neonatal sepsis [23, 28, 57]. Finally, the current study provided significant information through a single-centered retrospective study and an incidence trend. However, the contribution of every COVID-19 containment measures on the pattern of neonatal sepsis needs further investigation.

## Conclusion and recommendation

The study revealed that neonatal sepsis was prevalent at Jimma Medical Center and a common reason for NICU admission. The suitable software for detecting changes in trend, the joinpoint model, suggested a possible association between COVID-19 containment measures and decreased incidence and changing patterns of newborn sepsis. The incidence rate has been unsteadily declining, but the percentage change varied due to a multitude of confounding factors like health services, maternal, neonatal, environmental, or COVID-19 containment measures. More importantly, COVID-19 containment measures in the study center, such as mask use, avoidance of overcrowding, strict hand hygiene, visitor restriction and screening, and assigning senior staff for neonatal care, precisely enumerate newborn care. The study center intensively applied these measure with the pace of CIVID-19 pandemic and are thus potentially outfitted for the prevention and changing the patterns of neonatal sepsis. However, given the plethora of perplexing elements in the domain and the fragility in epidemiologic features of neonatal sepsis, its pattern with changes in health care facilities must still be continuously examined.

## Supporting information

S1 File Data analyzed in STATA software. (XLSX)

S2 File Data analyzed in Joinpoint regression. (TXT)

## Lists of abbreviations

MPC: Monthly Percent Change
COVID-19: Corona virus disease 2019
CR: Comparability Ratio
WHO: World Health Organization
JMC: Jimma Medical Center
CI: Confidence Interval
NICU: Neonatal Intensive Care Unit
